# Clinical severity grading of NF2-related schwannomatosis

**DOI:** 10.1186/s13023-024-03512-3

**Published:** 2025-01-06

**Authors:** Anna C. Lawson McLean, Denise Löschner, Said Farschtschi, Nora F. Dengler, Steffen K. Rosahl

**Affiliations:** 1 Department of Neurosurgery, University Hospital Jena, Am Klinikum 1, 07747 Jena, Germany; 2 Department of Neurosurgery, Helios Klinikum Erfurt, Erfurt, Germany; 3 Department of Neurology, University Hospital Hamburg Eppendorf, Hamburg, Germany; 4 Department of Neurosurgery, Charité - Universitätsmedizin, Berlin, Germany

**Keywords:** nf2-related schwannomatosis, Neurofibromatosis 2, Schwannomatosis, Grading

## Abstract

**Background:**

NF2-related schwannomatosis (NF2) is associated with various tumors of the central and peripheral nervous system. There is a wide range of disabilities these patients may suffer from and there is no validated clinical classification for disease severity. We propose a clinical classification consisting of three severity grades to assist in patient management.

**Methods:**

Patient records from 168 patients were screened for most common diagnoses with severe impact on everyday tasks, social interactions and life expectancy. Eight main categories were identified. One point was assigned to each category. Three severity grades were differentiated as follows: grade 1 (mild NF2): 0 points; grade 2 (moderate NF2): < 3 points; grade 3 (severe NF2): ≥ 3 points. This grading system was then evaluated with respect to inter-rater reliability and clinical significance.

**Results:**

The patients were grouped according to our new clinical grading system into grade 1 in 48% (*n* = 80), grade 2 in 43% (*n* = 72), and grade 3 in 10% of patients (*n* = 16). There was substantial inter-rater reliability between 3 raters with different levels of clinical experience (Fleiss’ kappa = 0.62). The severity grades correlated significantly with hospitalization, number of operations and dependency on implants (such as cochlear implant, auditory brain-stem implants or ventriculoperitoneal shunts).

**Conclusions:**

Clinical disease severity of NF2 patients is reflected in a simplified and rater-independent score with three grades. The score facilitates communication for medical personnel of varying experience and backgrounds, and adds a clinical tool to decision-making and research.

## Introduction

NF2-related schwannomatosis (NF2) is an autosomal-dominant genetic disease associated with multiple tumors of the central and peripheral nervous system along with further disease manifestations such as cataract and polyneuropathy. Concordantly, there is a wide spectrum of phenotypic expression. Most commonly, patients with NF2 will experience hearing deterioration due to bilateral vestibular schwannomas, which are present in 90–95% of cases. However, deficits may affect almost all sensory channels as well as motor and executive functions, depending on tumor location.

Historically, clinical severity has been categorized into the Wishart and Gardner subtypes. There is no formal definition of theses subtypes, but commonly the Wishart type is considered more severe with a young age of onset and rapid disease progression and additional clinical features like peripheral neuropathy. Truncating mutations have been found to cause this more severe subtype. The Gardner type, on the other hand, is associated with first diagnosis after 20 years of age and slow progression. However, this rough classification cannot encompass the complexity of the disease and does not take into account more recent developments, such as early diagnosis based on genetic testing. In a recent publication [1], we pointed out the need for an update of this classification system.

Genetics have been employed to establish grading systems for NF2. Ferner and colleagues proposed a genetic grading system based on 3 grades: (1) tissue mosaic; (2) classic NF2; and (3) severe NF2 [2]. Grade 1 (tissue mosaic) refers to patients in whom the same *nf2* mutation is present in multiple tumors but cannot be detected in all somatic cells. Grade 2 refers to classic NF2 cases including most types of *nf2* mutations, such as missense mutations, in-frame deletions and duplications as well as splice-site mutations. Grade 3 is reserved for the most severe genetic mutation of the *nf2* gene: a full truncating mutation of exons 2–13. The United Kingdom NF2 research group found correlations between genetic grading and several clinical and radiological parameters such as tumor load, age of disease onset, and age at loss of functional hearing [3].

Moreover, a disease-specific quality of life (QOL) score for NF2 was developed by Hornigold and colleagues [4]. The NFTI-QOL consists of eight items and was validated against the SF-36 and EuroQOL instruments. It has since been applied in several studies on patient-reported outcomes in NF2 [5–9]. Interestingly, in a 2014 publication by Ferner et al. the authors compare patient-reported quality of life with clinician-rated severity. However, clinician-rated severity in this publication vaguely refers to the Wishart (severe) and Gardner (mild) subtypes as well as an intermediate (moderate) subtype [5]. To our knowledge, there is no further published evidence of a validated clinical severity score for NF2.

Given the complexity of the disease and the clinical challenges arising during the disease and treatment course, a reliable clinical grading system is needed. A validated clinical grading system has the potential to facilitate interdisciplinary and inter-professional communication, decision-making as well as clinical research. To this end, we propose a clinical disease-severity grading system specific to NF2 based on clinical data from our supraregional NF2 center.

## Methods

Records of 168 patients with NF2 that have been treated at our specialized neurofibromatosis center over the past 10 years were retrospectively evaluated. All patient records were screened for functional diagnoses with an expected severe direct effect on social interactions / everyday tasks and life expectancy. These functional diagnoses were grouped into the following 8 categories:

- Loss of hearing on both ears.
- Severe visual impairment on both eyes (vision < 0.1) [10].
- Bilateral facial paralysis on at least one side (≥ House & Brackmann grade 3).
- Depression /anxiety disorder.
- Severe chronic pain / substance abuse due to pain.
- Immobility (patient not able to walk independently with or without a walking aid).
- Epilepsy.
- Malignancies (tumors with WHO grade 3 or 4).

In order to calculate disease severity, 1 point was provisionally assigned to each category. In a training set of ten randomly selected patients, different combinations of disease severity grades and associated scores were tested. According to these preliminary findings, a separation into 3 severity grades based on the following formula appeared to be the most useful classification:

Grade 1 (mild NF2): 0 points.

Grade 2 (moderate NF2): < 3 points.

Grade 3 (severe NF2): ≥ 3 points.

The validity of this separation into 3 grades was tested in a first round of assessment. The disease severity scores for 168 living patients with NF2 were independently determined by 3 individual raters, all of whom are employed at our specialist neurofibromatosis center. Rater 1 (SR) is a neurophysiologist and neurosurgeon with over 20 years of experience in the treatment of patients with NF2. Rater 2 (ALM) is a neurosurgical resident and has over 7 years of experience in the treatment of patients with NF2. Rater 3 (DL) is a board-certified neurosurgeon and has over 3 years of experience in the specialized treatment of patients with NF2. The current clinical disease severity grade was based on records from the latest visit to our institution by each patient. Inter-rater reliability was determined using Fleiss’ kappa.

In a second round of assessment, further retrospective data was obtained including number of doctor-patient contacts over the past years as well as number of operations and presence of implants. This data was used to determine the validity of the clinical disease severity grading by applying standard statistical tests such as Wilcoxon rank sum test, Chi-Square test and correlation analysis.

RStudio version 2023.03.1 + 446 was employed for all statistical analysis. A p-value < 0.05 was considered statistically significant.

This study was approved by the local institutional review board.

## Results

### Characteristics of patiens in our NF2-related schwannomatosis cohort

Our cohort consisted of 168 patients with NF2-related schwannomatosis and a median age of 40 years (range 4 to 92 years), 55% were female. 37% of patients had a loss of hearing in both ears, 2% of patients had severe visual impairment on both eyes, 6% had bilateral facial paralysis and 14% suffered from depression or anxiety disorder. 7% of patients developed chronic pain, 2% were immobile, 11% had epilepsy and 2% developed malignant tumors (Table 1).

**Table 1:** Patient characteristics of our cohort of 168 individuals

Patient characteristics	Number of patients (%)
**Female**	93 (55%)
**Median age**	40 years
**Loss of hearing**	63 (37%)
**Severe visual impairment**	3 (2%)
**Bilateral facial paralysis**	10 (6%)
**Depression/anxiety**	23 (14%)
**Chronic pain**	13 (7%)
**Immobility**	4 (2%)
**Epilepsy**	18 (11%)
**Malignancy**	3 (2%)

### Interrater variability of clinical grading

The clinical disease grade was determined by three individual raters of various experience. The results can be found in Table 2. The Fleiss’ kappa between 3 raters was 0.62 indicating substantial agreement according to Landis and Koch (1977) [11]. There was no case of disagreement between all three raters. In those cases where one rater differed from the others, consensus could be achieved after thorough discussion of the case.

**Table 2:** Disease severity grading by rater and final grading after thorough discussion. The Fleiss’ kappa for 3 raters was 0.62, which indicates substantial agreement

	Number of patients	Percentage of unanimous agreement	Percentage of agreement between at least two raters
**Grade 1**	80	77.5%	100%
**Grade 2**	72	56.5%	100%
**Grade 3**	16	61.5%	100%

### Clinical grading and correlations to doctor-patient contacts

80 patients were determined to be grade 1 (48%), 72 patients were grade 2 (43%), and 16 patients were grade 3 (10%).

In order to validate the clinical relevance of the grading system, correlations between grade and doctor-patient contacts (in-patient vs. out-patient) over the last year, number of operations within the last year and number of implants were assessed.

**Fig. 1:**
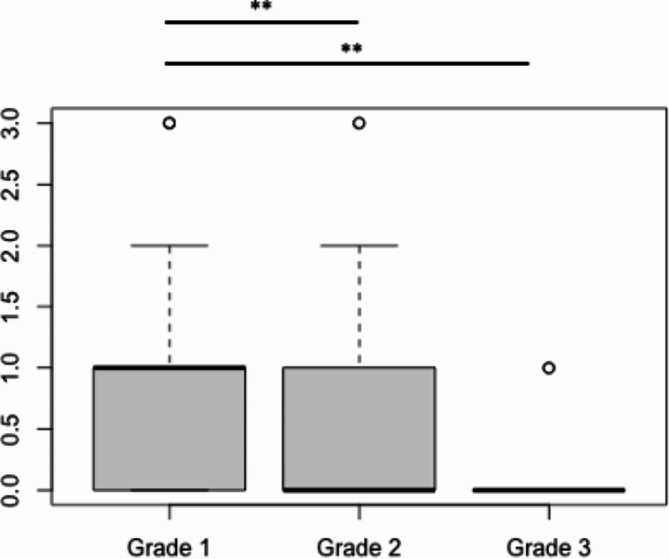
The median number of **annual out-patient contacts** ± standard deviation (SD) was 1 ± 0.7 for grade 1, 0 ± 0.5 for grade 2 and 0 ± 0.1 for grade 3, respectively. There was a significant difference between grade 1 and grade 2 as well as grade 1 and grade 3 (** *p* < 0.01)

**Fig. 2:**
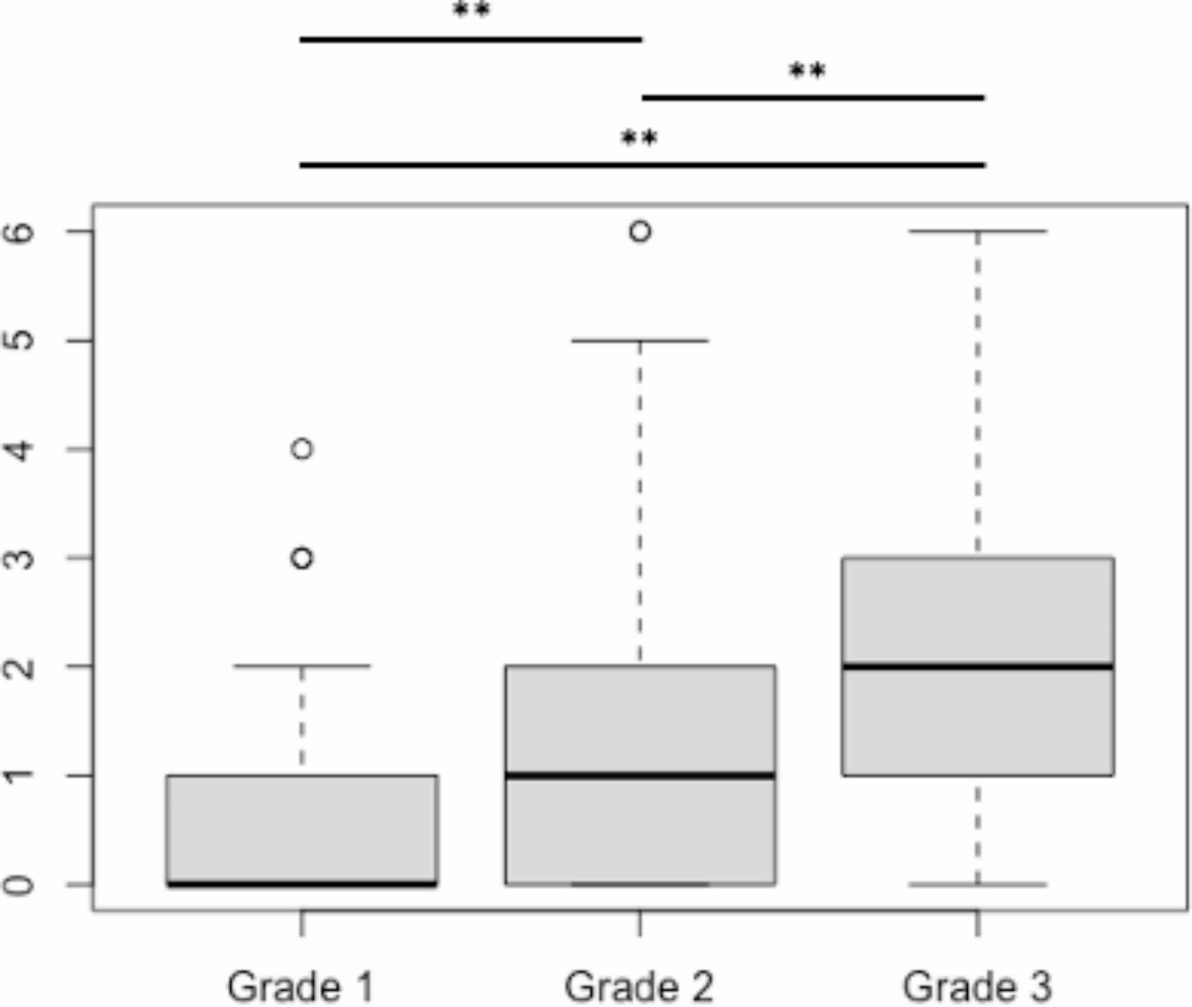
The median number of **annual admissions to the hospital** ± SD was 0 ± 0.6 for grade 1, 1 ± 1.5 for grade 2 and 2 ± 2.3 for grade 3, respectively. There was a significant difference between grade 1 and grade 2, grade 1 and grade 3 as well as grade 2 and grade 3 (** *p* < 0.01)

**Fig. 3:**
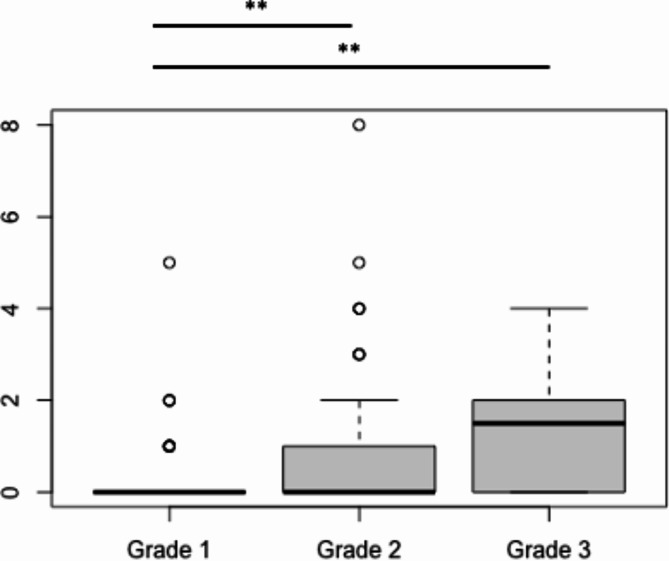
The median number of **annual operations** ± SD was 0 ± 0.3 for grade 1, 0 ± 1.0 for grade 2 and 1.5 ± 1.4 for grade 3, respectively. There was a significant difference between grade 1 and grade 2 as wells as grade 1 and grade 3 (** *p* < 0.01)

There was a significant difference between grade 1 and grade 2 as well as grade 3 regarding doctor-patient contacts and number of operations. Grade 1 patients were seen more often on an out-patient basis than grade 2 and grade 3 patients (Fig. 1), while grade 2 and grade 3 patients were more commonly admitted to the hospital on an in-patient basis (Fig. 2). In addition, the number of annual operations increased with grade (Fig. 3).

There was a moderate, but significant, correlation between grade and amount of auditory brain-stem implants (ABI) / auditory mid-brain implants (AMI) as well as between grade and amount of ventriculoperitoneal shunts (VP-shunts). Grade 1 patients had more cochlear implants (CI) than grade 2 and grade 3 patients, but there was no significant correlation (Table 3).

**Table 3:** Number of implants correlates with clinical grade

	Number of patients with CI	Number of patients with ABI/AMI*	Number of patients with VP-shunt
**Grade 1**	8/80	1/80	1/80
**Grade 2**	4/72	19/72	11/72
**Grade 3**	1/16	4/16	8/16
Cramer‘ s V (confidence interval)	0.08 (0.02–0.23)	0.36 (0.26–0.45)	0.43 (0.28–0.61)
p-value (chi-square)	0.576	2.472e-05	1.387e-07
* a total of 3 patients had AMIs (all grade 2)			

## Discussion

Preservation and restoration of function have increasingly moved into the focus of attention in the treatment of patients with neurooncological diseases, especially in those with hereditary genetic diseases such as NF2. Function is a priority and more often than not trumps radical tumor removal, especially in patients who have already lost major neurological functions. A clinical scoring system facilitates interdisciplinary and inter-professional communication and helps to identify those who are at risk of losing function and personal independence.

There are scoring systems for NF2 that focus on genetic severity and quality of life. Also, radiological imaging such as whole-body MRI may provide a comprehensive understanding of individual phenotypes of NF2 patients [12]. While these are undeniably important factors, a clinical severity grading appears to be crucial in order to identify high-risk patients and to aid clinical decision making such as follow-up intervals and necessity of surgical interventions.

In addition, the existing genetic severity score published by Ferner et al. [5] is to be considered a prognostic score. On the other hand, the clinical grading system proposed here is intended to be a dynamic tool, which should be re-assessed at each patient contact.

Purely radiological findings such a tumor load were not taken into account for the proposed scoring system as they only indirectly affect the patients’ life. Indeed, clinical experience tells us that a small intracanalicular vestibular schwannomas may cause more severe functional deficits than larger, but asymptomatic tumors. In the same way, there are many patients with NF2 who benefit greatly from hearing aids and implants and who experience no exclusion from verbal interactions. Therefore, only non-compensated hearing loss scores a point in our clinical severity classification. Similarly, we decided against including hydrocephalus in our clinical disease severity grading as these diagnoses are in most cases compensated for with respect to the patients’ quality of life and social functioning. Only if there is a direct functional effect (such as vision loss), these diagnoses will influence the disease severity score.

In our analysis, we identified statistically significant correlations between clinical grade and doctor-patient contacts as well as number of yearly operations and number of implants. We found that patients with grade 1 could mostly be managed on an out-patient basis while grade 2 and grade 3 patients were mostly seen on in-patient basis. In addition, grade 3 patients received more operations in one year than those of lower grades. The fact that grade 1 patients had the highest number of cochlear implants, and that the proportion of ABIs/AMIs and VP-shunts increased significantly in grade 2 and grade 3 patients, underlines their dependency on functionally and structurally corrective surgery.

The proposed grading system is intended to be a dynamic aid in the clinical work with patients with NF2. At each visit, the grade should be reassessed. In case of deterioration, further measures such as operative functional restoration, rehabilitation or educational classes (such as sign-language classes) should be taken into consideration.

With bevacizumab and brigatinib, medical treatment strategies have been established in NF2 besides surgical approaches; others are on the way in clinical trials. In the near future the individual risk stratification will not be of academical interest but will guide the therapeutical management. Questions about treatment onset, duration and combination with surgery and other agents remain open to this date. An clinical risk score could help to establish therapeutic guidelines for NF2.

We are aware that this grading system has several limitations. Each patient has their individual path and certain functional limitations may affect some more than others. The diversity among all patients and the variety of treatment options cannot be captured in 3 grades.

Data were collected retrospectively, which may introduce bias. Future prospective use of the scale will provide further confirmation of its utility and validation.

The term “immobility” is not well defined in the existing literature. The North American Nursing Diagnosis Association defines it as a state in which one “individual experiences or is at risk of experiencing limitation of physical movement” [13] and the term could be used in the context of paralysis, motor inertia, or fatigue. As we aimed to focus on functional outcomes rather than underlying pathologies, we allocated the point for all individuals who were not able to walk independently with or without a walking aid.

In addition, we believe, the patients’ age should be considered when assessing the physician-rated clinical severity score. Our cohort did not contain any patients with grade 2 or grade 3 who were below 20 years of age. However, it might be useful to add a “+”, i.e. grade 2 + or grade 3+, for young patients with a severe phenotype.

Overall, we consider this grading system a reliable tool to classify patients and to assist clinicians of various experience levels and various specialties to manage patients with this complex chronic neurogenetic disease. We will assess and test this grading system further in ongoing studies.

## Conclusions

We propose a novel clinical disease severity grading system specific to NF2 consisting of 3 grades (mild, moderate, severe). The grading system focuses on functional parameters and helps assess the effect of the disease on daily activities and the likelihood of hospitalization. It has substantial inter-rater reliability, highlighting its potential use among medical personnel of varying experience and backgrounds. This novel scoring system is intended to facilitate clinical decision-making, as well as clinical research on patient-reported outcomes and quality of life.

## Data Availability

Raw data for the utilized dataset are not publicly available to preserve individuals’ privacy under the European General Data Protection Regulation. However, the data that support the findings of this study are available from the corresponding author, ALM, upon reasonable request.
